# ROS, Klotho and mTOR in cardiorenal aging

**DOI:** 10.18632/aging.104209

**Published:** 2020-10-29

**Authors:** Nastaran Daneshgar, Dao-Fu Dai

**Affiliations:** 1 Department of Pathology, University of Iowa Carver College of Medicine, Iowa City, IA 52242, USA

**Keywords:** reactive oxygen species (ROS), Klotho, mTOR, kidney aging, cardiac aging

Aging is well known to predict decreased kidney function. The prevalence of chronic kidney diseases increases to more than double in the elderly population compared with that in the adult population. Our recent study in old C57Bl6 mice demonstrates increased glomerulosclerosis with compensatory glomerular hypertrophy, tubular atrophy, interstitial fibrosis, resulting in reduced cortical thickness [1]. These features closely resemble aging changes in human kidneys. As C57Bl6 mice do not develop spontaneous diabetes or significant hypertension with age, we did not find evidence of arteriosclerosis in aged mice. This contrasts with well-documented arteriosclerosis observed in kidneys from the elderly, whose prevalence of hypertension is more than 70% (in contrast to ~25% in young adults). Interestingly, proteomics analysis of glomeruli revealed an age-related decrease in several essential slit-diaphragm proteins, including nephrin and podocin. The reduction in these podocyte proteins may contribute to increased susceptibility to glomerulosclerosis in aging. Targeted tubular proteomics showed reduction of proteins involved in fatty acid oxidation and an increase in some glycolytic enzymes, suggesting a switch in substrate utilization in aged tubular epithelial cells, presumably due to declining mitochondrial function. Our data demonstrate that overexpression of glutathione peroxidase-1 (GPX-1), which reduces oxidative stress (especially in the mitochondria), significantly ameliorated many of the kidney aging phenotypes.

**Figure 1:**
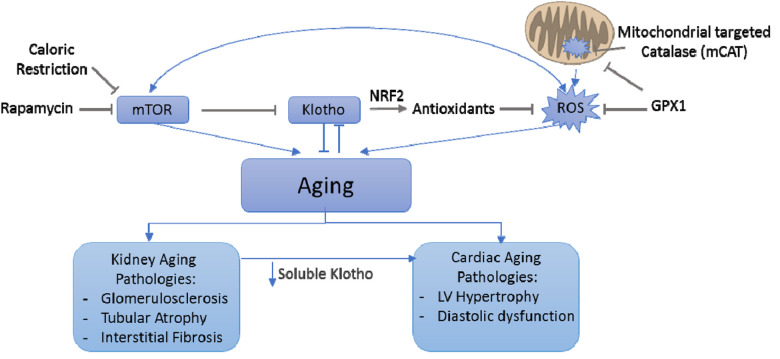
Interplay between mTOR/rapamycin, Klotko, ROS and mitochondria in both kidney and cardiac aging

Membranous α-klotho, predominantly produced by kidney tubular epithelial cells [2], is a co-receptor for FGF23, which regulates phosphate homeostasis. When membranous klotho is proteolytically cleaved by secretase, the resulting circulating / soluble α-klotho (sKL) acts as an aging suppressor hormone. Overexpression of Klotho in mice extends the life span through suppression of IGF1 signaling [2]. In contrast, mice homozygous for hypomorph (reduced function) klotho gene (Klotho^kl/kl^) displayed several “progeroid” phenotypes, including skin and muscle atrophy, hyperphosphatemia, osteoporosis, vascular calcification, and premature death [3]. Decreased circulating Klotho with age has been documented in both mice and humans. One proposed mechanism of aging phenotypes in the context of klotho deficiency is mediated by ROS, supported by increased oxidative damage in young Klotho^kl/kl^ mouse kidneys, like that found in kidneys from the old wild type mice. Despite the findings that antioxidant GPX1 attenuated several aging changes in the kidney, it did not protect against age-related decrease in klotho expression. These findings suggest that Klotho deficiency is likely upstream of ROS signaling (Figure 1), as reducing ROS by GPX1 did not affect the age-dependent decline in tissue Klotho expression [1]. Indeed, Klotho has been shown to attenuate oxidative damage through activation of transcription factor nuclear factor erythroid 2‐related factor 2 (Nrf2), a master regulator of the endogenous antioxidant system (Figure 1).

The anti-aging effect of rapamycin, an inhibitor of mammalian target of rapamycin (mTOR), may have been partly mediated by Klotho. Rapamycin upregulates Klotho expression, which leads to suppression of vascular calcification, whereas activation of mTOR suppresses Klotho gene expression and accelerates cellular senescence [4]. Since kidney is the main organ producing Klotho, chronic renal failure cause Klotho deficiency. This may also play critical roles in causing vascular calcification and uremic cardiomyopathy, leading to the well-known risk of cardiovascular mortality in chronic renal failure patients.

Our previous report showed that mice overexpressing catalase targeted to mitochondria (mCAT) ameliorated cardiac aging -- particularly age-dependent left ventricular hypertrophy and diastolic dysfunction. These findings emphasize the critical role of mitochondrial oxidative stress in cardiac aging [5]. We further showed that caloric restriction or rapamycin, presumably through inhibition of mTOR, rejuvenates cardiac health, better preserve youthful proteome (especially mitochondrial proteome) and reduces oxidative stress in the hearts of old mice [6]. Although the role of soluble Klotho in cardiac aging remains unclear, given its roles in modulating NRF2 and mediating some anti-aging effects of rapamycin, we propose that decreased soluble Klotho may represent an important mechanism of cardiovascular aging. This is supported by the following studies. Klotho deficient mice were more susceptible than wild-type mice to stress-induced pathological cardiac hypertrophy and remodeling. One of the mechanisms is through TRPC6 ion channel, which was upregulated by stress but inhibited by Klotho [7]. Furthermore, young heterozygous klotho-deficient mice undergoing 5/6 nephrectomy (to model accelerated chronic kidney disease) developed exaggerated cardiac hypertrophy resembling “uremic cardiomyopathy”, independent of hypertension or hyperphosphatemia. Restoration of soluble klotho by injection of sKL transgene ameliorated cardiac hypertrophy in chronic kidney disease (CKD) mice [8]. This finding suggests that the deficiency of soluble Klotho is a significant risk factor for CKD-related cardiovascular diseases, providing an example of cardiorenal crosstalk.

In summary, Klotho deficiency is a potential mechanism for age-related pathologies in both kidney and heart, making it a promising target to further our limited knowledge of aging and explore its application in ameliorating age-related phenotypes. The brief discussion above suggests a complex interplay between mTOR/ rapamycin, Klotho, ROS, and mitochondria in both kidney and cardiac aging (Figure 1).
